# Chagas Immunochromatographic Rapid Test in the Serological Diagnosis of *Trypanosoma cruzi* Infection in Wild and Domestic Canids

**DOI:** 10.3389/fcimb.2022.835383

**Published:** 2022-02-22

**Authors:** Esthefany S. Rodrigues, Gilbert Q. Santos, Marlon Vicente da Silva, Juliana H. S. Barros, Aline R. Bernardo, Rafaela L. Diniz, Nara M. Rubim, André L. R. Roque, Ana Maria Jansen, Edimilson D. Silva, Samanta C. C. Xavier

**Affiliations:** ^1^ Laboratory of Tripanosomatid Biology, Oswaldo Cruz Institute, Oswaldo Cruz Foundation (FIOCRUZ), Rio de Janeiro, Brazil; ^2^ Institutional Program for Initiation Scholarships in Technological Development and Innovation, Oswaldo Cruz Institute, Oswaldo Cruz Foundation (FIOCRUZ), National Council for Scientific and Technological Development (CNPq), Rio de Janeiro, Brazil; ^3^ Pedagogical Coordination Section, Army Complementary Training School and Salvador Military College, EsFCEx, Salvador, Brazil; ^4^ Diagnostic Technology Laboratory, Immunobiological Technology Institute (Bio-Manguinhos), Oswaldo Cruz Foundation (FIOCRUZ), Rio de Janeiro, Brazil; ^5^ Chagas Disease Translational Research Program, Fio-Chagas, Oswaldo Cruz Foundation, (FIOCRUZ), Rio de Janeiro, Brazil

**Keywords:** *Trypanosoma cruzi*, domestic and wild canids, dead-end and sentinel hosts, quimeric antigens, rapid test

## Abstract

*Canis lupus familiaris* (domestic dog) represents a reliable sentinel for the occurrence of a well-established transmission cycle of *Trypanosoma cruzi* among wild mammals in the surroundings and, consequently, where the risk of human infection exists. Serological diagnosis is the chosen method to identify *T. cruzi* infection in dogs that, in Brazil, rarely present positive parasitological tests. The use of recombinant chimeric parasitic antigens results in a sensitive and specific serological diagnostic test in contrast to the use of crude *T. cruzi* antigens. Our objective was to evaluate the Chagas/Bio-Manguinhos Lateral Flow Immunochromatographic Rapid Test (Chagas-LFRT) for the diagnosis of *T. cruzi* infection in domestic dogs and the potential of application of this diagnostic platform to wild canid species. Two recombinant proteins (IBMP-8.1 and IBMP-8.4) that displayed the best performance in the enzyme immunoassay (ELISA) in previous studies were tested in a platform with two diagnostic bands. A panel of 281 dog serum samples was evaluated: 133 positive for *T. cruzi* by serological diagnosis, including 20 samples with positive blood cultures belonging to different discrete typing units (DTUs); 129 negative samples; and 19 samples from dogs infected by other trypanosomatids: *Leishmania infantum*, *Trypanosoma rangeli*, *Trypanosoma caninum* and *Crithidia mellificae*, in addition to samples infected by *Anaplasma platys*, *Dirofilaria immitis* and *Erlichia* sp. that were employed to evaluate eventual cross-reactions. We also evaluated the Chagas-LFRT to detect *T. cruzi* infection in 9 serum samples from six wild canid species. We observed that the intensity pattern of the bands was directly proportional to the serological titer observed in IFAT. The sensitivity was 94%, the specificity was 91% according to the ROC curve, and the defined cutoff was an optical density of 4.8. The agreement obtained was considered substantial by the kappa analysis (84%). From *T. cruzi* positive hemoculture samples, 88.9% were positive by Chagas-LFRT. The test was efficient in recognizing infections by five of the six *T. cruzi* DTUs. Cross-reactions were not observed in infections by *L. infantum*, *T. rangeli*, *T. caninum* and *D. immitis*; however, they were observed in sera of dogs infected by *Crithidia mellificae*, *Anaplasma* sp. and *Erlichia* sp. A strong reaction was observed when serum samples from wild canids were submitted to the Protein A affinity test, confirming its applicability for these species. This test will allow rapid preventive actions in areas with high risk to the emergence of Chagas disease in a safer, reliable, low-cost and immediate manner, without the need for more complex laboratory tests.

## Introduction

*Trypanosoma cruzi* (Chagas, 1909), the etiologic agent of Chagas disease (CD), is a flagellate protozoan of the Trypanosomatidae family that is widely distributed in nature, occurring from the southern United States to southern Argentina and Chile. It is capable of infecting almost all tissues of hundreds of species of mammals to which it is transmitted by hematophagous hemiptera of the subfamily Triatominae, known as kissing bugs (barbeiros, vinchuca, bicudos and many other regional terms in Latin America) (Galvão et al., 2003; Noireau et al., 2009; Jansen et al., 2015). *T. cruzi* is a taxon that presents expressive intraspecific diversity whose interpretation has been challenging scientists since Carlos Chagas. Currently, seven genotypes or discrete typing units (DTUs) are recognized in the species, namely, TcI-TcVI, in addition to a genotype described as Tcbat. The latter, initially associated with Chiroptera (Marcili et al., 2009), has already been detected in humans (Guhl et al., 2014; Ramírez et al., 2014a).

Trypanosomiasis by *T. cruzi* is primarily a wild enzooty. Its transmission in nature takes place within a complex trophic network, in which each animal species plays a different role, in space and time, regarding its maintenance and infective competence (Jansen et al., 2015; Jansen et al., 2020), resulting in distinct enzootic scenarios. In other words, each region is unique and has a specific transmission network, which needs to be understood and known so that one can recognize areas of epidemiological risk and correctly guide health agents and local residents (Roque et al., 2008).

Dogs represent the first domestic *T. cruzi* hosts studied by Carlos Chagas and are among the first experimental models used by him (Jansen et al., 2017). Among the different species of mammals, *Canis lupus familiaris* (domestic dog) has a significant role in the epidemiology of *T. cruzi* because it can act as a bioindicator. This is because this taxon is demonstrably capable of acting as a competent sentinel, signalizing the transmission of the parasite among wild mammals (Travi, 2018). Unlike other countries, domestic dogs in Brazil rarely present high parasitemia; that is, they display low infective potential (Xavier et al., 2012; Brandão et al., 2020). A proposal from our group that has received increasing attention from the Brazilian Health Authorities is the longitudinal serological survey of domestic and peridomestic mammalian species to determine the prevalence and/or incidence of *T. cruzi* infection (Jansen et al., 2017).

Serological diagnosis is the method of choice to assess the spread of the wild transmission cycle of *T. cruzi* and, consequently, define areas where the risk of human disease occurs. However, serological tests such as the indirect immunofluorescence reaction test (IFAT) and enzyme immunoassay (ELISA) often use a complex mixture of parasitic antigens, which can be related to false negative or false positive results (Santos et al., 2018). This fact is due to the possibility of cross-reactions of *T. cruzi* antigens with those other trypanosomatids, such as *Leishmania* sp. or other *Trypanosoma* species because they share common epitopes. With the development of recombinant DNA technology, several bacterial and eukaryotic gene expression systems allow the production of parasite antigens in large quantities, with a high degree of purity and standardized quality (Silveira et al., 2001; Foti et al., 2009). Thus, studies have shown that to improve diagnostic accuracy, the selection of more specific antigenic fragments for *T. cruzi* would be effective (Silveira et al., 2001; Camussone et al., 2009; Marchi et al., 2011; Santos et al., 2016; Santos et al., 2017; Peverengo et al., 2018; Santos et al., 2018; Daltro et al., 2019; Del-Rei et al., 2019; Leony et al., 2019; Silva et al., 2020). Chimeric proteins consist of antigenic sequences of the parasite, presenting a series of epitopes, which increases the diagnostic sensitivity (Santos et al., 2021). The IBMP proteins (IBMP-8.1, IBMP-8.2, IBMP-8.3 and IBMP-8.4) were evaluated their potential for diagnosing *T. cruzi*. The *T. cruzi* proteins whose antigenic regions (IBMP muti-epitope antigens) were used to construct the chimeric antigens are described in the [Supplementary Material of the study by Santos et al. (2017)].

Silva et al. (2020) showed that the lateral flow immunochromatographic rapid test (LFRT) for the diagnosis of *T. cruzi* in humans, using the chimeric proteins IBMP-8.1 and IBMP-8.4, is extremely efficient, and our idea was to expand the use of this test for the diagnosis of this parasite in domestic dogs and others species of wild canids in its original format. The use of two chimeric proteins increases the diagnostic potential of the rapid test as it increases the amount of available *T. cruzi* epitopes. This is because the two chimeric proteins used have different epitopes due to their different compositions. The rapid test results are quick, easy to perform and interpret, do not require laboratory infrastructure, and use a small amount of biological material (serum, plasma or whole blood). Additionally, there is no need for equipment and specific knowledge to carry it out. The use of the test will allow rapid preventive actions to be taken in places with or without notification of Chagas disease (Sánchez-Camargo et al., 2014). Consequently, it will bring benefits mainly to locations where access to a more complex laboratory test is limited.

The recombinant antigens were formatted in a rapid immunochromatographic test using either *Staphylococcus aureus* protein A or *Streptococcus pyogenes* protein G as the gold-labeled reagents for the visualization of the precipitin band formed between the immunoglobulin (Ig) G-specific antibodies and the recombinant antigen immobilized on the nitrocellulose stripe used in the test. Despite the fact that most rapid immunochromatographic tests are formatted with protein A, the rationale to test both protein A and protein G was based on the fact that these microbial molecules bind with different affinities and specificities to immunoglobulins of various species, including dogs (Frohman et al., 1979; Nilsson et al., 1982; Bjorck and Kronvall, 1984; Costa et al., 2003).

Here we need to consider that: 1) establishing a diagnosis based on only one serological test is not secure enough. Two tests are always required, one being confirmatory; 2) the screening of dogs (not to mentioning wild animals) in the field is extremely advantageous, due to the speed of the response of the test, which already allows the immediate adoption of some mitigation measures. Biological material is a difficult to be maintained in field conditions, and bad storage conditions may even result in its loss. Also, the subsequent communication of results to the examined community constitutes a challenge. There are still many places in Brazil without internet access or mail making the communication of the results a difficult task.Insufficient access to diagnosis is yet a major issue to diagnose *T. cruzi* infection. Therefore, there is a need for more practical diagnostics and/or diagnostic algorithms that better suit the demands and field conditions and are indispensable to prevent the reemergence of CD in vulnerable regions of Brazil with similar epidemiological characteristics. Such diagnostics should be made available not only to easily detect *T. cruzi* infection, including different DTUs and mixed infection but also to avoid crossed reaction by other trypanosomatid infections (Gállego et al., 2020). In this study, it was evaluated the performance of recombinant chimeric antigens (IBMP-8.1 and IBMP-8.4) for the detection of anti-*T. cruzi* IgG antibodies in dog sera using Chagas-LFRT.

## Materials and Methods

### Ethical Statements

All procedures performed with wild and domestic animals received authorization from IBAMA (wild canids) and followed protocols approved by FIOCRUZ’s Animal Use Ethics Committee (P0007-99; P0179-03; P0292/06; L0015-07; L-050/2016; LW-81/12).

### Protein A Affinity Test for Wild and Domestic Canids

Serum samples from species of the order Carnivora were submitted to the Protein A affinity test to validate the use of the Chagas/Bio-Manguinhos Lateral Flow Immunochromatographic Rapid Test (Chagas-LFRT) for domestic and wild canids. The species tested were *Canis lupus familiaris* (n = 4), *Canis lupus* (n = 2), *Chrysocyon brachyurus* (n = 1), *Lycalopex vetulus* (n = 2), *Lycaon pictus* (n = 1), *Speothos venaticus* (n = 1) and *Cerdocyon thous* (n = 3). These samples were submitted to the Bio-Manguinhos Canine Visceral Leishmaniasis DPP LVC^®^ Rapid Test kit, which also uses protein A, and the intensity in the control band marking was used to classify the strength of the interaction between the antibodies of the dog species and this protein, regardless of the marking of diagnostic bands. The affinity of the interaction with Protein A of these samples was classified as strong (++++), medium (+++), low (++) and very low (+).

### Wild and Domestic Canids Serum Samples

**Figure 1:**
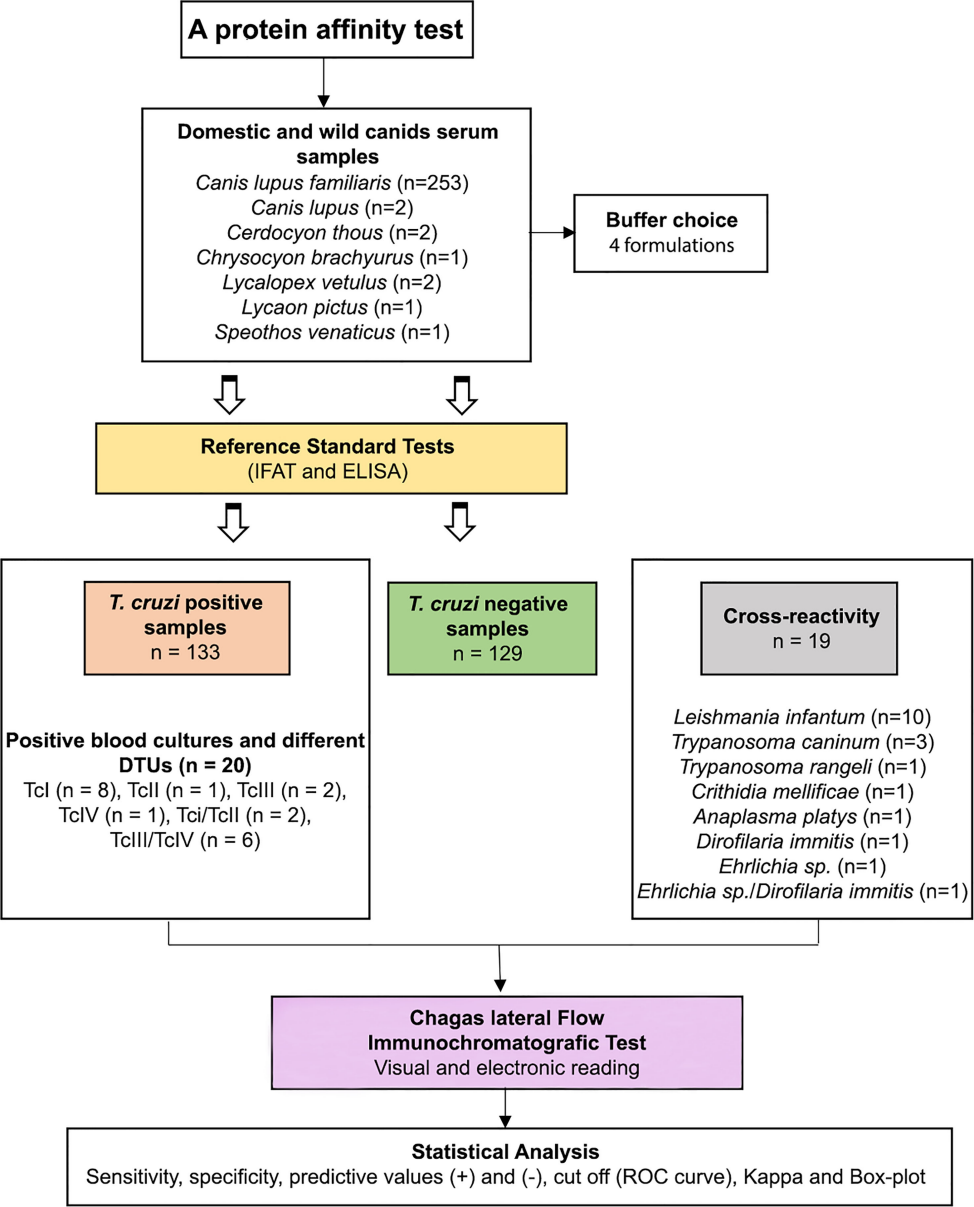
Scheme showing the serum samples from wild and domestic canids with previous serological diagnosis (IFAT and ELISA) selected to compose the panel of positive and negative samples and serum samples with different parasitic infections used in the Chagas lateral flow immunochromatografic test (Chagas-LFRT).

We used 253 serum samples from *Canis lupus familiaris* and nine serum samples from six species of wild canid to detect *T. cruzi* infection: European wolf, *Canis lupus* (n = 2), *Cerdocyon thous* (n = 2), *Lycalopex vetulus* (n = 2), *Lycaon pictus* (n = 1), *Speothos venaticus* (n = 1) and *Chrysocyon brachyurus* (n = 1). *C. l. familiaris* samples were collected in distinct scientific expeditions to different regions of Brazil by the Laboratory of Trypanosomatids Biology (LABTRIP) and by the Laboratory of Wild Mammals Reservoir Biology and Parasitology, both from the Oswaldo Cruz Institute – FIOCRUZ/Rio de Janeiro between 2007 and 2019. Wild canid samples were obtained from the Zoo Park Foundation of São Paulo and from studies conducted in the southeastern Goiás state municipality of Cumari (Brandão et al., 2020) (**Table 1** and **Figure 1**). This material is kept at -20°C in the LABTRIP serum bank. Sera were aliquoted and each aliquot thawed only once.

**Table 1:** Distribution of wild and domestic canids in the Brazilian biomes based on molecular characterization of parasite populations isolated in blood cultures, the total number examined by blood cultures and serological tests.

Biomes	State	Municipality	Wild and/or Domestic Canids	Positive IFAT and ELISA (N)	Negative IFAT and ELISA (N)	Positive hemocultures (N)	Parasite characterization
**Amazon Forest**	Acre	Feijó	*Canis lupus familiaris*	14	71		
		Marechal Thaumaturgo	*Canis lupus familiaris*	2		DTU TcIV (1)	
		Rodrigues Alves	*Canis lupus familiaris*	12	8	DTU TcI (3)	
	Pará	Abaetetuba	*Canis lupus familiaris*	1		DTU TcI (1)	
		Belém	*Canis lupus familiaris*	1	1		
		Cesarea	*Canis lupus familiaris*	4			
		Curralinho	*Canis lupus familiaris*	2			*Trypanosoma rangeli*(1)
		Ilha do Combú	*Canis lupus familiaris*	11	1	DTU TcI (2)	
		Monte Alegre	*Canis lupus familiaris*	4		DTU TcI/TcII (2)	
		São Domingos do Capim	*Canis lupus familiaris*	2			
**Caatinga**	Ceará	Carnaubal	*Canis lupus familiaris*	2			
		Croatá	*Canis lupus familiaris*	4			
		Ibiapina	*Canis lupus familiaris*	12			
		Jaguaruana	*Canis lupus familiaris*	2			
		Redenção	*Canis lupus familiaris*	1			
		Viçosa do Ceará	*Canis lupus familiaris*	4			
	Pernambuco	Ibimirim	*Canis lupus familiaris*	1	1	DTU TcI (1)	
	Piauí	São Raimundo	*Canis lupus familiaris*		2		
**Cerrado**	Goiás	Cumari	*Canis lupus familiaris*	5	6	DTU TcIII/TcV (2)	
			*Cerdocyon thous*	1		DTU TcIII/TcV (1)	
			*Lycalopex vetulus*	2		DTU TcIII/TcV (2)	
	Tocantins	Ananás	*Canis lupus familiaris*	1			
		Araguatins	*Canis lupus familiaris*	8	25		
		Nazaré	*Canis lupus familiaris*	1			
**Atlantic Rain Forest**	Espírito Santo	Guarapari	*Canis lupus familiaris*		1*	DTU TcII (1)	
	Rio de Janeiro	Angra dos Reis	*Canis lupus familiaris*		11		
		Mangaratiba	*Canis lupus familiaris*				*Crithidia mellificae* (1)*, Trypanosoma caninum* (1)
		Rio de Janeiro	*Canis lupus familiaris*				*Anaplasma platys* (1)*, Ehrlichia* sp. (1), *Dirofilaria immitis* (1)*, Trypanosoma caninum* (2), *Ehrlichia* sp./*D. immitis* (1)
	São Paulo	Zoo Park Foundation of São Paulo	*Canis lupus*	2			
			*Canis lupus familiaris*	2			
			*Cerdocyon thous*	1			
			*Chrysocyon brachyurus*		1		
			*Lycaon pictus*	1			
			*Speothos venaticus*		1		
**Pampas**	Rio Grande do Sul	São Borja	*Canis lupus familiaris*	2			*Leishmania infantum* (9)
**Pantanal**	Mato Grosso do Sul	Urucum	*Canis lupus familiaris*	28		DTUs TcI (1), TcIII (2), TcIII/TcV (1)	*Leishmania infantum* (1)
				**133**	**129**	**20**	**19**
		

*Sample with positive hemoculture and negative IFAT (1/20) and ELISA, but positive in Chagas-LFRT.

DTU, Discrete Typing Unit

Serum samples with previous serological diagnosis (IFAT and ELISA) were selected to compose the panel of positive (n = 133) and negative (n = 129) samples. The serologically positive samples were those with positive IFAT (titer ≥ 1/40) and ELISA (optical density ≥ 0.200). Within the panel of positive samples, samples with positive blood cultures (n = 20), including *T. cruzi* isolates characterized by different DTUs TcI (n = 8), TcII (n = 1), TcI/TcII (n = 2), TcIII (n = 2), TcIV (n = 1), and TcIII/TcV (n = 6), were included to validate the test based on the recognition of the different genotypes of the parasites deposited in the Coleção de *Trypanosoma* de Mamíferos Silvestres, Domésticos e Vetores, and COLTRYP/Fiocruz (Oswaldo Cruz Foundation, Rio de Janeiro, RJ, Brazil). To compose the panel of serum samples with different parasitic infections (n = 19), we used samples diagnosed as *Leishmania infantum* (n = 10), *Trypanosoma caninum* (n = 3), *Trypanosoma rangeli* (n = 1), *Crithidia mellificae* (n = 1), *Anaplasma platys* (n = 1), *Dirofilaria immitis* (n = 1), *Ehrlichia* sp. (n = 1) and a sample with mixed infection by *Ehrlichia* sp./*Dirofilaria immitis* (n = 1) (**Table 1** and **Figure 1**). All samples of dogs serum used in the cross-reaction panel were subjected to immunofluorescence antibody test (IFAT) and ELISA to diagnose *T. cruzi* and *Leishmania infantum* infection and those positive for *T. cruzi* were excluded from the analysis.

### Chagas^®^ Bio-Manguinhos Rapid Test (Chagas-LFRT)

The Chagas rapid test works with lateral flow immunochromatographic labeling, employing a combination of a protein conjugated with colloidal gold particles and *T. cruzi* antigens (recombinant proteins IBMP-8.1 and IBMP-8.4) bound to a solid phase (nitrocellulose membrane) (Silva et al., 2020). The sample to be analyzed is applied to a specific area of the plastic holder (cassette), followed by the addition of a running buffer. The buffer provides lateral flow of released components, promoting the binding of antibodies to antigens. Four different formulations of running buffer with and without blocking were tested, with modifications to the buffer formulation: high, medium or low blocking of nonspecific reactions in the tests and TR DPP LVC^®^ canine visceral leishmaniasis from Bio-Manguinhos kit buffer.

**Figure 2:**
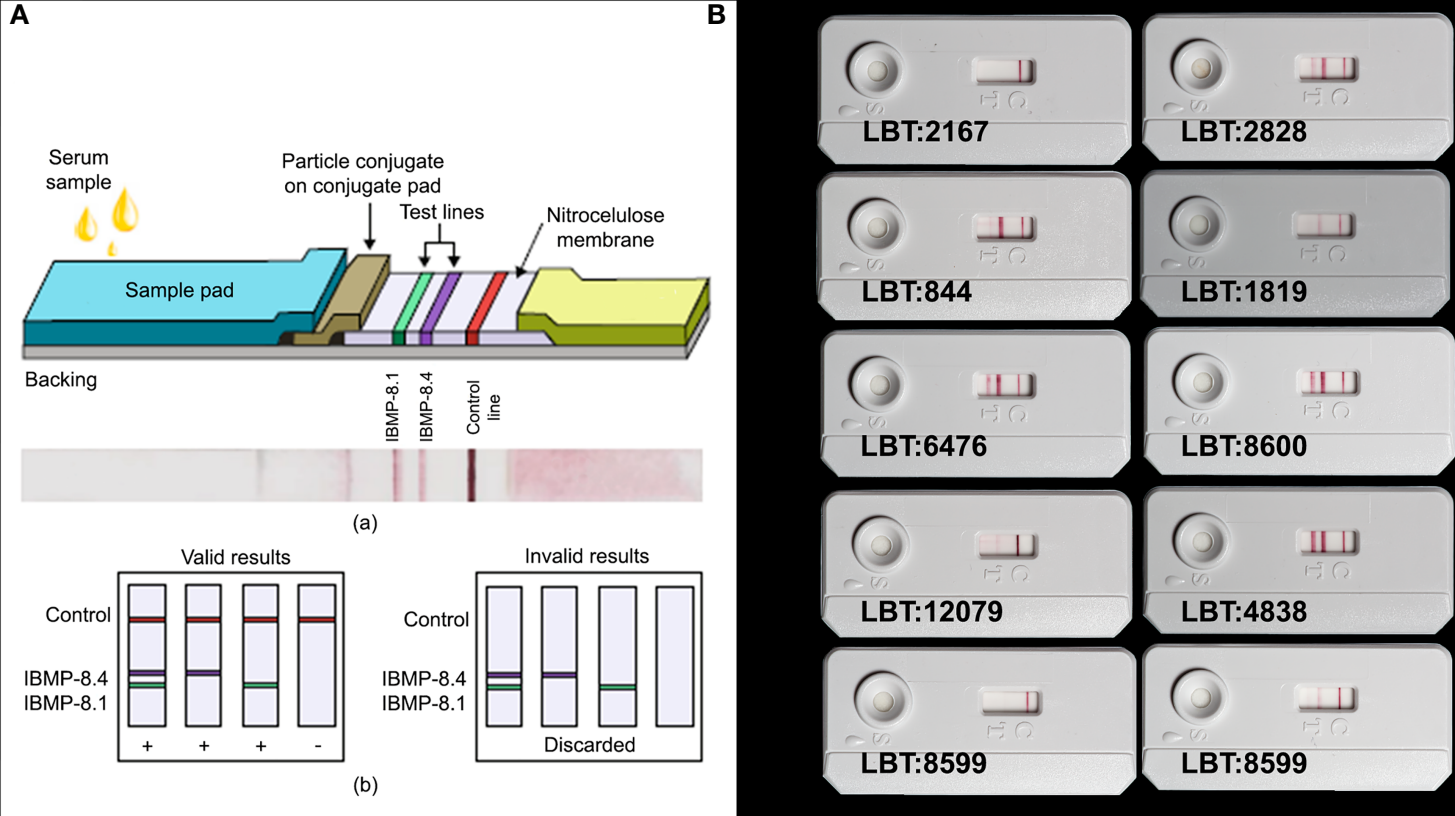
**(A)** Lateral flow assay (LFA) device. (a) Schematic illustration of the composition of the LFA strip for the detection of IgG anti-*Trypanosoma cruzi* in samples employing the IBMP-8.4 and IBMP-8.1 chimeric antigens and photographic image of an LFA opened device after loading *T. cruzi*-specific IgG antibodies. (b) Schemes of the results expected for valid and invalid results yielded by LFA. Copyright^©^ Silva et al., 2020, https://doi.org/10.1155/2020/1803515. **(B)** *Canis lupus familiaris*: LBT 2167 (-), LBT 2828 (+), LBT 844 (+; DTU TcI), LBT 1819 (+; DTU TcI/TcII), LBT 6476 (+; DTU TcIII), LBT 8600 (+; DTU TcIII/V), LBT 12079 (+; DTU TcIV); *Canis lupus*: LBT 4838 (+); LBT 8599: sample from a domestic dog with seroconversion event (2009 and 2016).

To reveal the reaction, a protein A system conjugated to colloidal gold was used. The use of this type of conjugation basically privileges reactions with IgG because it makes use of the extraordinary affinity of protein A with the Fc region (crystallizable fragment) of IgG. Antibodies present in the sample bind to specific proteins conjugated to colloidal gold. In case a sample is positive, the “immunoconjugate” complex migrates on the nitrocellulose membrane, being captured by the fixed antigens in the test area and producing two purple/pink lines. In the absence of specific anti-*T. cruzi* antibodies, the purple/pink lines do not appear in the test area. In all cases, the sample continues to migrate across the membrane, producing a purple/pink line in the control area, which demonstrates proper functioning of the reagents (**Figure 2A**).

The test was performed by applying 5 µL of sample (serum, plasma or whole blood) to the sample well followed by the addition of three drops of running buffer using a dropper bottle with a measuring nozzle. This mixture migrates by capillarity, eluting the conjugate (protein A conjugated to colloidal gold) to the test area where there are the antigens obtained through collaboration with the Proteomics and Protein Engineering Laboratory of the Carlos Chagas Institute - ICC/Fiocruz/PR, which provided two recombinant chimeras named IBMP-8.1 (21 KDa) and IBMP-8.4 (40 KDa). These chimeric proteins were expressed in *Escherichia coli* and purified by chromatography (Santos et al., 2016; Santos et al., 2017). Excess nonreaction-related components migrate to the other end of the tape where they are retained. The reading was carried out between 10 and 15 minutes, counted with the addition of running buffer, and incubated at room temperature. All inputs used in standardization are from the Chagas Rapid Test/Bio-Manguinhos (https://www.bio.fiocruz.br/index.php/br/produtos/reativos/testes-rapidos/teste-rapido-chagas) for Chagas disease produced by Bio-Manguinhos (Silva et al., 2020).

### Agreement Between Visual and Automated Reader

Visual reading of the recognition densities of the test and control bands was performed by two observers, as well as with a rapid lateral flow test reader to define the sensitivity and specificity parameters of the test to equate the inherent subjectivity of the human eye when reporting results in samples with low titers of antibodies. This type of device was developed in collaboration with the Carlos Chagas Institute-ICC/PR, Paraná Molecular Biology Institute-IBMP/PR and the Paraná Technological University-UTFPR/PR. Visual reading was standardized as follows: N1= strong negative; N2=weak negative; P1= positive of weak intensity; P2= positive of medium intensity and P3= positive of strong intensity (**Figure 2B**). The automated reader expresses the results quantitatively (ranging from 0 to 100) in optical density (OD), performing the reading for both diagnostic bands and the control band. These values were used to define the cutoff by the ROC curve.

### Statistical Analysis

A statistical summary of the data, a box-plot graph and a cluster analysis were performed to determine the Chagas rapid test cutoff point for classification of data collected by the automated reader of the IBMP-8.1 and IBMP-8.4 chimeric antigens by the matrix of confusion, which is intended to evaluate the performance of a binary classifier. The test performance (sensitivity and specificity) was evaluated format in single or duplex using the IBMP-8.1 and IBMP-8.4 proteins, separately and together to the serological diagnosis of *T. cruzi* infection wild and domestic Canids. The cut off, sensitivity, specificity and accuracy values were established by determining the largest area under the ROC curve (receiver operating characteristic) (Swets, 1988). The ROC curve is a statistical and graphical method for determining the best cutoff point for a diagnostic test. The highest point on the curve, corresponding to the upper left angle of the graph, represents 100% sensitivity and 0% false positives (d = 0); in this case, the ideal value for a diagnostic test, called the gold standard. Samples were separated by IFAT serological titers to generate the ROC curve and calculate sensitivity, specificity and cutoff values. Cohen’s kappa (K) analysis was used to determine the strength of agreement between the reference standard tests (IFAT and ELISA) and the Chagas LFRT, which was interpreted as weak agreement (< 0), slight agreement (0.01-0.20), fair agreement (0.21-0.40), moderate agreement (0.41-0.60), substantial agreement (0.61-0.80), and near perfect agreement (0.81-0.99) (Cohen, 1960). All data analyses were performed using RStudio software version 1.2.5033 and R version 3.6.3, and a p-value under 5% (p < 0:05) was considered significant.

## Results

### Protein A Affinity Test for Wild and Domestic Canids

In the Protein A affinity test of samples from different species, all canids (wild and domestic), a strong interaction was demonstrated for the species *Canis lupus familiaris*, *Canis lupus*, *Chrysocyon brachyurus*, *Lycalopex vetulus*, *Lycaon pictus*, *Speothos venaticus* and *Cerdocyon thous*. This result allowed the choice of *Canis lupus familiaris* for the standardization of the Chagas rapid test for *T. cruzi*, in addition to this animal being considered an important sentinel of infection in different areas of study. Among the four formulations of running buffer with different types of blocking tested, the buffer selected was the original of the commercial kit of Chagas Rapid Test (with blocking) because it presented greater intensity in the labeling of the chimeric proteins IBMP-8.1 and IBMP-8.4. The selected buffer showed a high intensity recognition profile, visually classified as P3 for the two chimeric proteins and for the test control band.

### Chagas^®^ Bio-Manguinhos Rapid Test in the Diagnosis of *T. cruzi* Infection in Dogs

**Figure 3:**
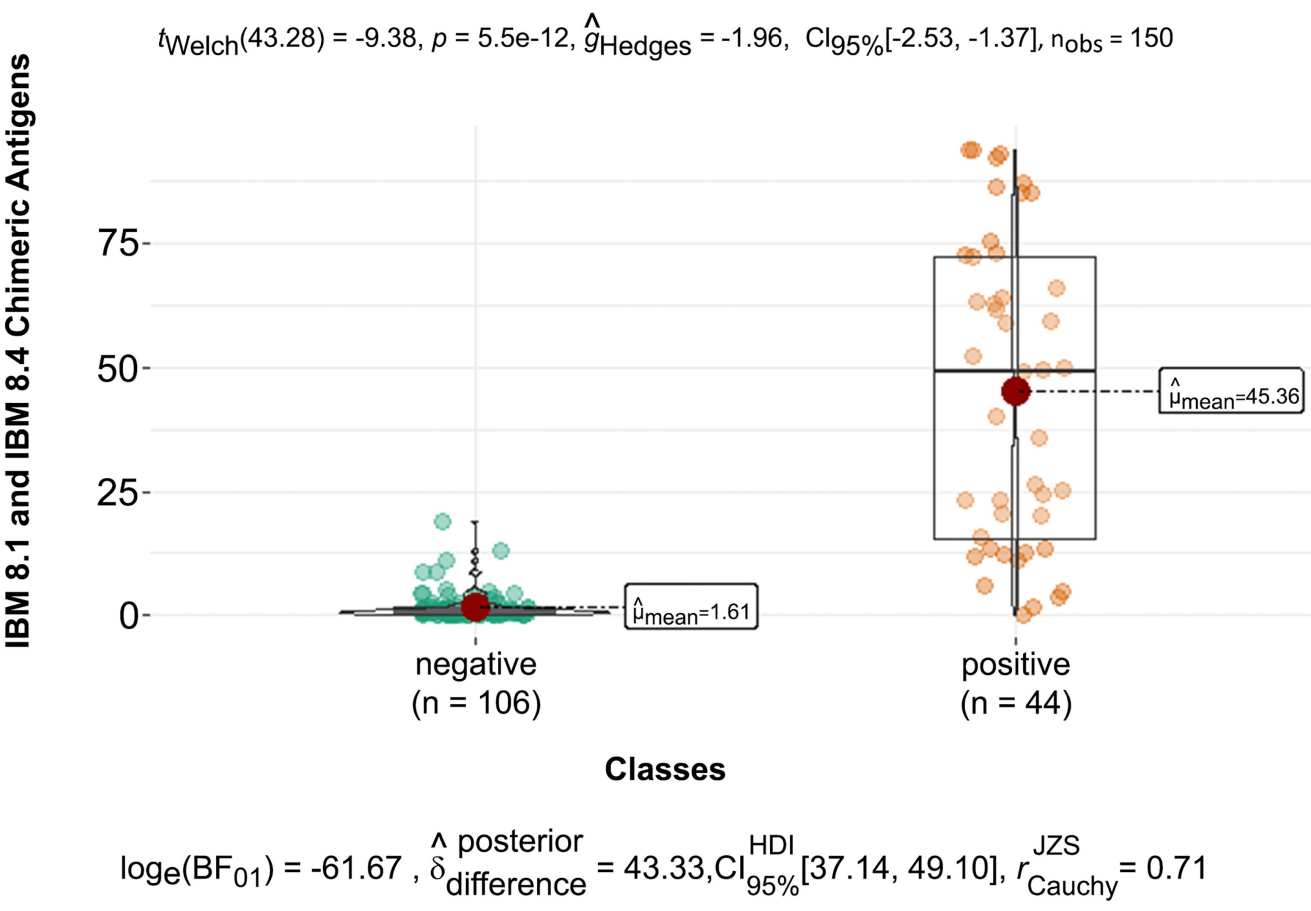
BoxPlot with the distribution of mean values of optical densities and mean values of negative and positive samples from dogs infected by *Trypanosoma cruzi* against the chimeric antigens IBMP-8.1 and IBMP-8.4.

**Figure 3** shows the mean optical density values (OD 1.61) in serum samples from dogs with negative serological diagnosis. The mean of the optical density values of the samples from dogs infected by *T. cruzi* was 45.36 against the chimeric antigens IBMP-8.1 and IBMP-8.4. Among the positive samples considering the cutoff point OD ≥ 4.8, the IBMP-8.1 antigen was positive in 68% and the IBMP-8.4 antigen in 60% of the samples tested, and an agreement of 45% was observed between the IBMP-8.1 and 8.4 antigens in the positive samples against *T. cruzi* infection. This result highlights the importance of combining two antigens in a single test to increase the test sensitivity in the diagnosis of *T. cruzi* in dogs. Among the negative samples, only one (OD 5.1) reacted against the IBMP-8.1 antigen and means (OD 1.49), and four were false positive against the IBMP-8.4 antigen (OD ≥ 4.8 and ≤ 10.9) and the mean of the negative samples (OD 1.18).

**Figure 4:**
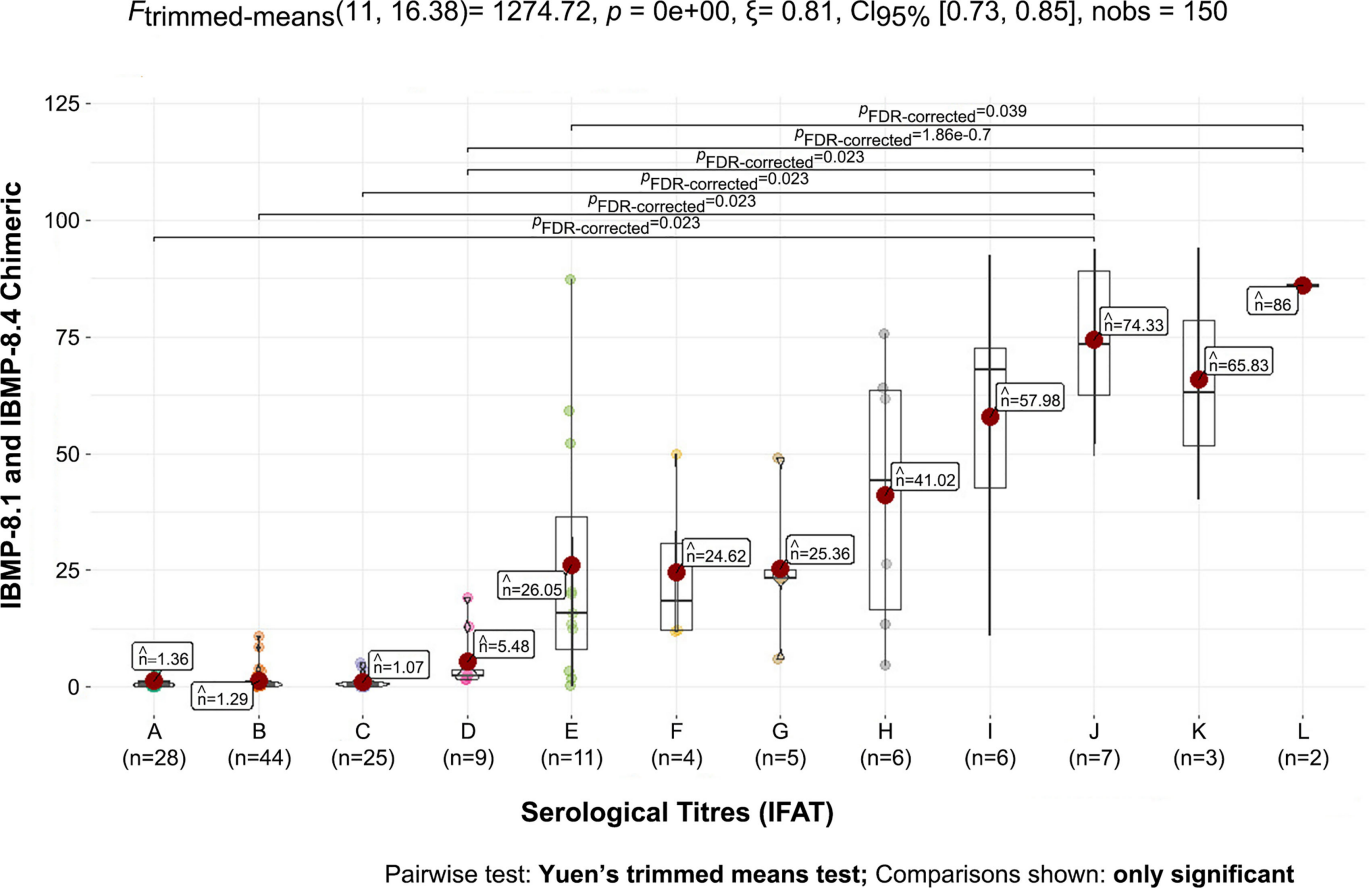
Mean of negative and positive samples from dogs infected by *Trypanosoma cruzi* against the chimeric antigens IBMP-8.1 and IBMP-8.4. Number of samples (n) and mean optical densities obtained by serological titer (A - negative, B - 1/10, C - 1/20, D - 1/40, E - 1/80, F - 1/160, G - 1/320, H - 1/640, I - 1/1280, J - 1/2560, K – 1/5120, L – 1/10240).

The optical densities obtained by the negative and positive samples were directly proportional to the serological titer by IFAT. The automated reading of the Chagas rapid test showed a pattern in which the optical density increased as the IFAT serological titer increased, mainly demonstrated by the means (**Figure 4**).

### Visual and Automated Reader

Visual analysis showed high accordance with the band intensities of the IBMP-8.1 and IBMP-8.4 chimeric antigens according to the automated reade*r* device. Negative titers showed total absence of color in 93.8%, classified as N1 and N2. Among the panel of negative samples by IFAT (titer ≤ 1/20), the observer rated was 78 (N1) and 13 (N2), 2 (N2/P1) and 4 (P1) false positive samples. In the panel of positive samples (titer ≥ 1/40), the observer classified as follows: 13 (P1), 15 (P2), 15 (P3) and 10 samples were classified as false negatives: 3 (N1) and 7 (N2). The intermediate titers (1/40 to 1/320) had a medium intensity in the test zone lines, mainly diagnosed as P1 (10), P2 (9) and P3 (1), as the higher titers (1/640 to 1/20480) showed the strongest intensities in the marking of chimeric proteins, with the most prevalent visual diagnosis being positive with strong intensity P3 (14), P2 (6) and P1 (3). The visual classification did not present a significant difference in the classification based on the optical densities of the automated reader in relation to the cut off 4.8 for positive and negative samples; that is, the visual classification can replace the reader’s classification by Kappa analysis (**Figure 2B** and **Table 2**).

**Table 2:** Relationship of agreement between visual reading according to serological titers given by IFAT and Chagas rapid test positive P1, P2 and P3 and negative N1 and N2, response patterns.

Serological titer	Visual reading agreement (%)	Visual reading variation (n)				
		N1	N2	P1	P2	P3
**Negative**	26/28 (92.8%)	**23**	3	2	0	0
**1/10**	43/44 (97.7%)	**38**	5	1	0	0
**1/20**	24/25 (96.0%)	**17**	7	1	0	0
**1/40**	6/9 (66.6%)	2	**4**	**3**	0	0
**1/80**	8/11 (72.7%)	1	2	**4**	3	1
**1/160**	4/4 (100%)	0	0	**2**	**2**	0
**1/320**	5/5 (100%)	0	0	1	**4**	0
**1/640**	5/6 (83.3%)	0	1	1	**2**	**2**
**1/1280**	6/6 (100%)	0	0	2	1	**3**
**1/2560**	7/7 (100%)	0	0	0	2	**5**
**1/5120**	3/3 (100%)	0	0	0	1	**2**
**1/20480**	2/2 (100%)	0	0	0	0	**2**

The numbers in bold indicate the largest number of samples read visually between the classifications.

### Positive Blood Culture and DTUs Recognition Pattern

Of the samples with positive blood cultures, 90% (18/20) had a positive result through visual and automated reading in the Chagas-LFRT. Samples from wild and domestic canids infected with the DTUs TcI, TcII, TcIII, TcIII/TcV and TcIV were considered positive by the test. Only two samples from domestic dogs with positive blood cultures (DTU TcI), with titers of 1/40 and 1/80, had a negative result in Chagas-LFRT (**Figure 2B**).

### Cross-Reaction With Trypanosomatids and Other Parasites From Canids

Regarding the samples infected with other trypanosomatids and other parasites from domestic canids, all samples from dogs infected by *L. infantum*, *T. rangeli*, *T. caninum* and *D. immitis* presented negative results in the Chagas-LFRT. However, positive samples for the parasites *Anaplasma platys*, *Crithidia mellificae*, *Ehrlichia* sp. and a sample with a mixed infection of *Ehrlichia* sp. and *D. immitis* showed weak positive results for *T. cruzi* in the rapid test (P1). The test was able to evaluate 78.9% (15/19) of the samples infected by other dog parasites as negative (**Table 1**).

### Statistical Analysis

**Figure 5:**
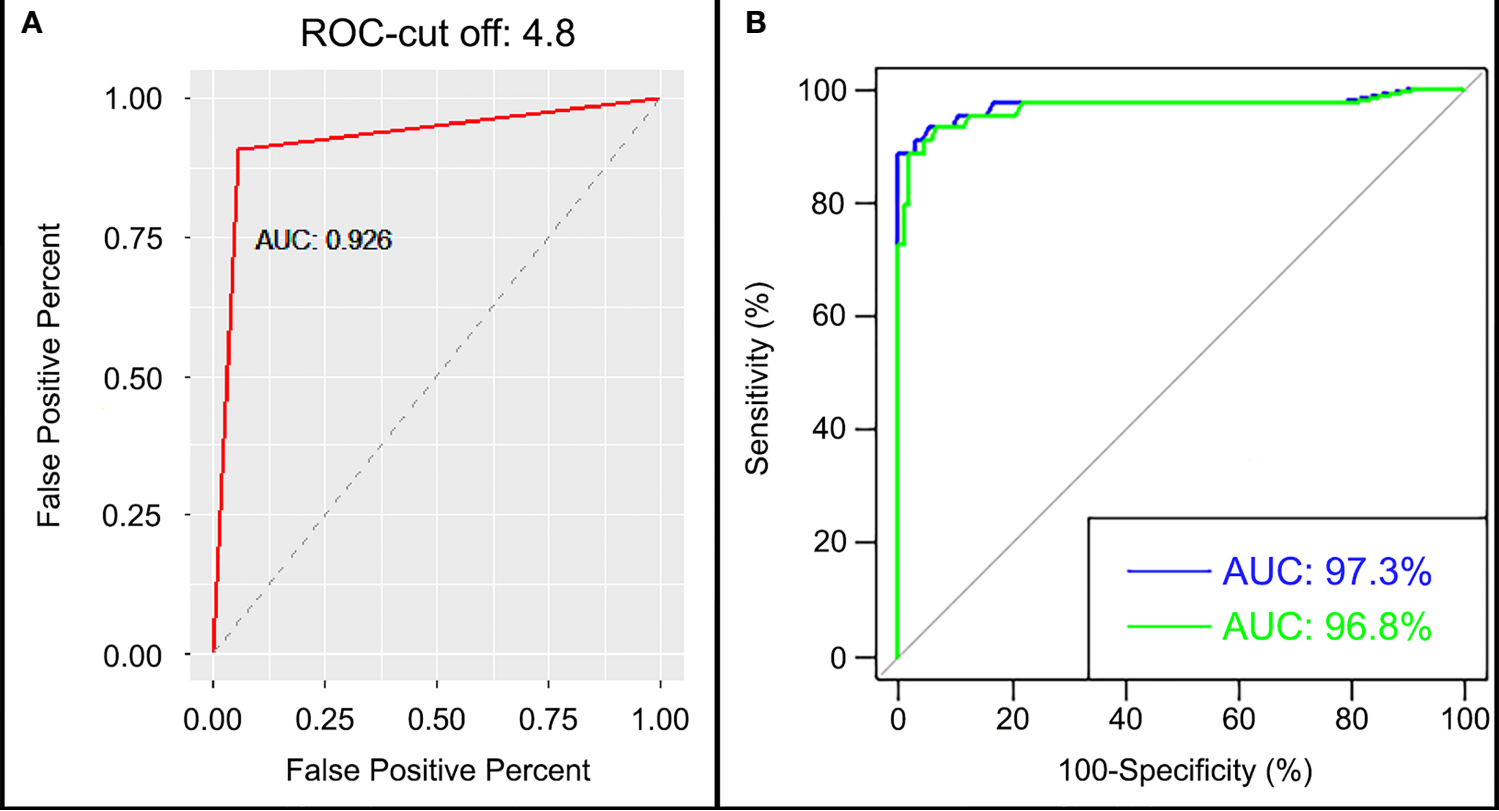
**(A)** Receiver operating characteristic (ROC) curve and area under the curve (AUC) of cutoff point value 4.8 and **(B)** AUC of cutoff (-) >1/20 cutoff (-) >1/40.

When we only use one single of the chimeric proteins the sensitivity and specificity was lower (BMP-8.1: sensitivity 92.5% and specificity 85.6% or IBMP-8.4: sensitivity 75% and specificity 83.3%), already the duplex test format (with two chimeric proteins, IBMP 8.1 and IBMP8.4). The chimeric proteins IBMP-8.1 and IBMP-8.4 using Chagas-LFRT had a sensitivity of 94% and a specificity of 91%, increasing the diagnostic power of the test when used the two chimeric proteins to the serological diagnosis of *T. cruzi* infection wild and domestic Canids. Five cutoff points were tested and evaluated (2.3, 3.7, 4.8, 5.9 and 8.7), and the cutoff point was considered an optical density of 4.8 and an area under the curve (AUC) value of 92.6% (**Figure 5A** and **Supplementary Figure S1**). The ROC curve presented an AUC of 97.3% for the cutoff point by IFAT of >1/20 and 96.8% for samples >1/40 in IFAT (**Figure 5B**). In the Kappa test, the coefficient was 0.84 (elevated), accuracy was 0.90, predictive positive values was 0.96 and negative values was 0.87 between the IFAT and the automatic reader of the Chagas rapid test (**Table 3** and **Supplementary Figure S1**).

**Table 3:** Summary and statistical parameters, considering cutoff 4.8 for the chimeric antigens IBMP-8.1 and IBMP-8.4, confidence interval (95% CI).

Parameters	statistic	Lower	Upper
**Apparent prevalence**	0.69	0.61	0.77
**True prevalence**	0.71	0.63	0.78
**Sensitivity**	0.94	0.88	0.98
**Specificity**	0.91	0.78	0.97
**Positive predictive value**	0.96	0.9	0.99
**Negative predictive value**	0.87	0.74	0.95
**Positive likelihood ratio**	10.38	4.07	26.45
**Negative likelihood ratio**	0.06	0.03	0.14
**Kappa**	0.84	0.68	1.00

## Discussion

One of the main bottlenecks in monitoring parasitosis in wild and even domestic animals is the small number of simple and feasible diagnostic methods to be applied in the field. In this sense, the test that we are proposing fills an important gap both in the feasibility and in the spectrum of animal taxa in which trypanosomatid infection can be diagnosed. Establishing access to diagnostics for wild animals species is of utmost epidemiological and epizootiological importance as the health of all animals (including humans) is profoundly interdependent and this awareness is always increasing as clearly stated in the One Health perspective (Jansen et al., 2015).

At present, most Chagas disease occurs in a new epidemiological profile in which *Trypanosoma cruzi* transmission occurs independently of the domiciliation of triatomines. A new approach to control measures is necessary, as the measures previously applied, in this case, the use of insecticides to control species of intradomiciliary triatomines, does not fit the current epidemiological reality. Targeted control of reemerging transmission can be achieved by improved understanding of *T. cruzi* in canine populations. Castillo-Neyra et al. (2015) suggest that dogs may be useful sentinels to detect reinitiation of transmission following insecticide treatment. A correlation between dogs with positive serology to *T. cruzi* and proximity to an infected triatomine (distance ≤50 m) was observed, suggesting that dogs may be useful sentinels to detect reinitiation of transmission following insecticide treatment (Castillo-Neyra et al., 2015).

The use of sentinel dogs has proven to be appropriate for any area that wants to assess the risk of human disease regardless of biome or cultural habits. Diagnosis using rapid tests is absolutely suitable for field conditions. It will enable large-scale diagnosis of areas of transmission of *T. cruzi* and, consequently, of risk for human disease. This work is perfectly aligned with the National Agenda of Priorities in Health Research, which textually recommends the development of new models and approaches for the surveillance of adverse health events and emerging diseases and the development of new technologies for the epidemiological surveillance of health problems, including specific forms of monitoring and scenario studies. Integration of canine *T. cruzi* blood sampling into existing interventions for zoonotic disease control (e.g., rabies vaccination programs) can be an effective method of increasing surveillance and improving the understanding of disease distribution (Castillo-Neyra et al., 2015).

The classical bottleneck has always been to preserve biological samples in field conditions that are often rather precarious besides transporting samples to the central level, as well as dispose of personnel sufficiently trained to carry out conventional methods of serological diagnosis on a large scale. Fast and easy tests to be performed have been increasingly sought after in regard to diagnosis. In this study, we performed an evaluation of the performance of the rapid lateral flow immunochromatographic test (Chagas-LFRT) for the serological diagnosis of *T. cruzi* infection in *Canis lupus familiaris* using two chimeric proteins (IBMP-8.1 and IBMP-8.4) as antigens. In this way, a quick decision-making process that will shape the epidemiological surveillance actions associated with the transmission of the parasite is made possible. Point-of-care tests are applicable in different sectors and bring short-term results in healthcare facilities and remote locations to complex diagnostic methods (Silva et al., 2020). Here, the use of a rapid test for dogs was validated, which has already been tested and approved for the diagnosis of *T. cruzi* infection in humans (Silva et al., 2020), optimizing its production and expanding its application in the field. Our results show that the test was sensitive to detect seroconversion, enhancing its application as a first measure to monitor the presence of the parasite, which indicates active transmission in the area, attested by seroconversion events, despite the low rate of positive parasitological tests in dogs from Brazil (Xavier et al., 2012; Brandão et al., 2020).

Dogs are animals that live together and accompany humans since they are still in the hunter-gatherer stage. In an evolutionary trade-off, dogs guaranteed safety, and humans, in turn, guaranteed dog food and shelter. In several cultures, dogs were used for different purposes, including prewarming the feet, hunting, guarding, adorning, sports and even a source of protein. Additionally, dogs are very common domestic animals and are easily traceable, and annual rabies vaccination campaigns as well as leishmaniasis control programs have resulted in the training of health service personnel in handling and collecting blood from these animals (Xavier et al., 2012; Daltro et al., 2019).

Dogs participate in the transmission cycle of *T. cruzi* in different ecoepidemiological scenarios, in addition to being a common animal in all areas, regardless of environmental and climatic conditions, which are not limiting for its occurrence. In Brazil, the pattern of *T. cruzi* infection in dogs is more related to the maintenance of the parasite in low/absence parasitemia, which results in low importance for the transmission of the parasite (Xavier et al., 2012). The plasticity of *T. cruzi* in nature ensures that it is transmitted in multiple environmental characteristics that vary in space and time, which is supported by the richness and diversity of wild mammals and vectors. In these multifactorial scenarios, the dog can be used as a sentinel for *T. cruzi* presence. Thus, the diagnosis of *T. cruzi* in domestic dogs is extremely important, as the dog’s role in the transmission of *T. cruzi* has been described as a dead-end host and sentinel of infection in the wild environment (Roque and Jansen, 2008). Areas where the dog’s infection is ≥ 30% signal the presence of a wild transmission cycle occurring close to the dog’s circulation areas, indicating the risk of Chagas disease cases (Xavier et al., 2012). As this test proved to be highly viable for the diagnosis of the parasite in dogs in our study, its use for detecting *T. cruzi* infection in dogs will allow area monitoring in real time, signaling areas with transmission of the parasite in the wild environment and perhaps also undetected human cases. Thus, accurate situational diagnosis is still in the field.

As *T. cruzi* is a parasite with high genetic variability, there is a significant difference in the performance of commercial serological tests related to the antigens used (Dopico et al., 2019). Therefore, the serological diagnosis of *T. cruzi* infection is quite complex due to the lack of reference methods. Furthermore, methods using parasite DNA are viable only in the initial phase of infection and are not capable of attesting to parasite viability (Daltro et al., 2019; Leony et al., 2019; Silva et al., 2020). Due to this situation, the World Health Organization (WHO) recommends the use of two tests to complete the diagnosis of *T. cruzi* infection (WHO Expert Committee, 2002). The diagnosis of the parasite is even more complicated in regard to wild and domestic canids due to the lack of validated tests. The antigenic matrices currently used can lead to low specificity and a high rate of cross-reaction with other trypanosomatids and other dog parasites (Leony et al., 2019).

Costa et al. (2003) evaluated both IgG binding proteins in rapid immunochromatographic test kits for the diagnosis of canine visceral leishmaniasis. The tests were assembled with either *Leishmania infantum* recombinant antigens K39 or K26 and with either gold-labeled *Staphylococcus aureus* protein A or *Streptococcus pyogenes* protein G. The test using recombinant antigens K39 or K26 produced results that were only slightly superior to protein A than to protein G (no significant differences were observed), possibly because this minor difference in sensitivity between these 2 tests is due to the slightly higher affinity that protein A has over protein G for dog IgG (Miele and Krakowka, 1981; Peng et al., 1991). Additionally, our test that relies on gold-labelled *Staphylococcus aureus* protein A conjugate that may be easily adapted to other wild species thus, optimizing large-scale surveys. This is especially important in investigation of disease outbreaks. Therefore, our test is opening the possibility of the diagnosis not only of *T. cruzi* in dogs but also of other trypanosomatids species infections in other species that domestic dogs.

Chimeric antigens provide the diagnosis with a greater number of parasite epitopes, thus reducing false-negative results when compared to the use of nonrecombinant antigens (Dopico et al., 2019). The capacity of the rapid test to detect all *T. cruzi* DTUs that circulate in Brazil, also suggested by Santos et al. (2017) and Dopico et al. (2019), is an important characteristic. In fact, in Brazil, there is a predominance of TcI and TcII DTUs, which are also the most frequent DTUs in wild carnivores and dogs (Rocha et al., 2013; Jansen et al., 2018; Brandão et al., 2020), but other DTUs may also be observed at minor rates. As an example, DTUs TcIII and TcIV have already been described in infected dogs in Brazil (Brandão et al., 2020). The chimeric antigens IBMP-8.1 and IBMP-8.4 in the rapid test platform are presented as tools capable of overcoming limitations imposed on other antigens and are also successful in other platforms investigated in previous studies, such as indirect ELISA and liquid microarray (Santos et al., 2016; Santos et al., 2017; Santos et al., 2018; Del-Rei et al., 2019; Leony et al., 2019; Silva et al., 2020).

Leony et al. (2019) used three reference strains (Colombian, Y and Berenice), known to have TcI and TcII DTUs, for experimental infection of domestic dogs with *T. cruzi*. This study was successful in diagnosing the parasite using the chimeric proteins through ELISA. Here we emphasize that, in addition to expanding the recognition panel of DTUs different from those analyzed by Leony et al. (2019), our study showed the possibility of using a rapid test platform with chimeric proteins to detect *T. cruzi* in naturally infected domestic and wild dogs.

The breadth of the serological profile of the samples used in our study, which included samples with recent and older infections and with different serological titers, indicates that the cutoff point found in our study for dog samples was 4.8, indicating the high diagnostic accuracy of the chimeric proteins IBMP-8.1 and IBMP-8.4 for dog and wild carnivore infections. A sample of serum from a domestic dog from Guarapari (Espírito Santo) was included in two panels: panel of negative samples in the serological diagnosis (IFAT and ELISA) with titer of 1/20 in the RIFI, below the cut-off point and panel of positive samples in the parasitological examination. This type of phenomenon is not uncommon and suggests an initial infection, when the immune response is not yet established.

Leony et al. (2019) found high sensitivity for four chimeric proteins, including two tested here (IBMP-8.1, IBMP-8.2, IBMP-8.3 and IBMP-8.4), in the diagnosis of *T. cruzi* infection in domestic dogs using ELISA. However, Leony et al. (2019) found cross-reactivity to IBMP-8.1 for at least one of all tested parasites (anaplasmosis, babesiosis, dirofilariosis and ehrlichiosis), with the exception of other trypanosomatids. IBMP-8.1, in Chagas-LFRT, also cross-reacted with other parasites, with positive results for anaplasmosis, ehrlichiosis and *Crithidia mellificae*. However, unlike the study by Leony et al. (2019), this antigen did not react with dirofilariosis in the rapid test Chagas. The chimeric protein IBMP-8.4 did not cross-react on the ELISA platform, but in the rapid test, it reacted with *Ehrlichia* sp. This can be explained by the difference number of epitopes provided by IBMP-8.4 compared to IBMP-8.1 (Del-Rei et al., 2019). Herein, we propose the use of Chagas-LFRT for the serological diagnosis of *T. cruzi* infection in dogs as a screening test in the field to monitor the area and that a second confirmatory test (IFAT and/or ELISA) has to be used for individual diagnosis.

We did not observe cross-reactions with the main trypanosomatids described infecting dogs (*L. infantum, T. caninum* and *T. rangeli*), and these results show that the rapid test for *T. cruzi* also proves to be a useful and viable tool in places where there is spatial and temporal overlapping of transmission by other trypanosomatids. Daltro et al. (2019) evaluated the cross-reactivity of chimeric proteins in the diagnosis of *T. cruzi* through ELISA in human samples with American Tegumentary Leishmaniasis and Visceral Leishmaniasis, finding practically negligible reactivity. Daltro et al. (2019) state that the use of chimeric parasitic antigens can reduce the cost of diagnosis, since there would be no need to repeat tests on the same sample, suggesting their use where *Leishmania* sp. and *T. cruzi* are coendemic.

There are hundreds of wild mammal species in which *T. cruzi* is capable of infecting and sampling, and diagnosing infection in free-living wild fauna is still a huge challenge. One of the biggest obstacles is the lack of diagnostic improvement. As a result of the anthropocentric view of health, only products for serological diagnosis of humans and animals of economic interest are commercially found. Thus, the potential to perform the diagnosis through Protein A or G linked to colloidal gold in a rapid test will multiply the application, since a specific antibody is not necessary to reveal the antigen-antibody reactions as in conventional serological tests. It will bring benefits mainly for the diagnosis of wild mammal fauna.

We propose here the spatiotemporal monitoring of infection in domestic dogs as an environmental diagnostic tool through Chagas-LFRT to be used as a first measure in the identification of areas where there is a potential risk of transmission of *T. cruzi* and the presence of the vector. The sole use of Chagas-LFRT can be used for an environmental diagnosis, while a second confirmatory test has to be employed for the individual diagnosis. Domestic dogs are animals that are easy to handle, in addition to having spatial and temporal traceability. The collection of blood from these animals does not require large infrastructure and cost, especially with the use of the rapid test for *T. cruzi*, a point-of-care technology that is fast and easy to handle. From this, Chagas-LFRT proved to be sensitive for use as a first environmental diagnostic tool for the presence of *T. cruzi* for early monitoring of the risk of new human cases.

## Conclusion

Our main contribution was to validate and expand the use of the rapid Chagas test, which was developed in Bio-Manguinhos for the diagnosis of Chagas disease in humans (Silva et al., 2020), for field diagnosis of *T. cruzi* infection in domestic dogs and to evaluate its potential of application to wild canid species. Our motivation was to implement in the field work routine, a quick test that is easy to perform (point-of-care exam), which does not require technical training and is not dependent on a complex laboratory infrastructure for its execution, as in the conventional diagnostic tests available (ELISA and IFAT). One of the great advantages is to have one same type of test that can be used under the same conditions for the diagnosis of *T. cruzi* infection in humans, domestic and wild dogs.

The surveillance system as a whole will benefit because it will have in its hands a specific and reliable rapid test for “in loco” diagnosis of infected dogs and proximity or presence of infected triatomines, that is, the signalling of an expressive enzootic cycle of *T. cruzi* transmission near human dwellings. Chagas-LFRT will allow rapid diagnosis in a safer, reliable and low-cost manner without the need for more complex laboratory tests. The detection of “hot areas” of enzootic transmission detected by the test will streamline the decision-making process by allowing quick mapping of target areas for implementation of prevention measures.

## Data Availability Statement

The datasets presented in this article are readily available. Requests to access the datasets should be directed to Samanta Xavier, samanta@ioc.fiocruz.br.

## Ethics Statement

The animal study was reviewed and approved by IBAMA (wild canids) and followed protocols approved by FIOCRUZ’s Animal Use Ethics Committee (P0007-99; P0179-03; P0292/06; L0015-07; L-050/2016; LW-81/12). Written informed consent was obtained from the owners for the participation of their animals in this study.

## Author Contributions

Conceptualization, SX, AJ, ES, and NR. Formal analysis, SX, ER, NR, AJ, and ES. Funding acquisition, SX. Investigation, SX, AJ, and AR. Methodology, SX, ER, GS, MS, JB, AB, RD, NR, ES, and AJ. Project administration, SX, NR, AJ, and ES. Resources, AB, RD, ES, and NR. Supervision, SX, ES, NR, and AJ. Visualization, SX, AJ, AR, NR, and ES. Writing original draft, ER, SX, JB, AJ, NR, and AR. Writing—review and editing, ER, SX, AJ, and AR. All authors have read and agreed to the published version of the manuscript.

## Funding

SCCX has received financial support from CNPq (MCTIC/CNPq No. 28/2018 - Universal, process number 422489/2018-2) and from Faperj ARC_2016 - Auxílio ao Pesquisador Recém-contratado (E-26/010.000249/2017).

## Conflict of Interest

The authors declare that the research was conducted in the absence of any commercial or financial relationships that could be construed as a potential conflict of interest.

## Publisher’s Note

All claims expressed in this article are solely those of the authors and do not necessarily represent those of their affiliated organizations, or those of the publisher, the editors and the reviewers. Any product that may be evaluated in this article, or claim that may be made by its manufacturer, is not guaranteed or endorsed by the publisher.

## Supplementary Material

Supplementary Figure S1 Receiver operating characteristic (ROC) curve, area under the curve (AUC) and statistical summary of cutoff point values (2.3, 3.7, 4.8, 5.9 and 8.7).
