# Supplementary figures and images for: Chagas Immunochromatographic Rapid Test in the Serological Diagnosis of Trypanosoma cruzi Infection in Wild and Domestic Canids
Source: Front Cell Infect Microbiol. 2022 Feb 22;12:835383. doi: 10.3389/fcimb.2022.835383 (PMC8902141; doi:10.3389/fcimb.2022.835383)

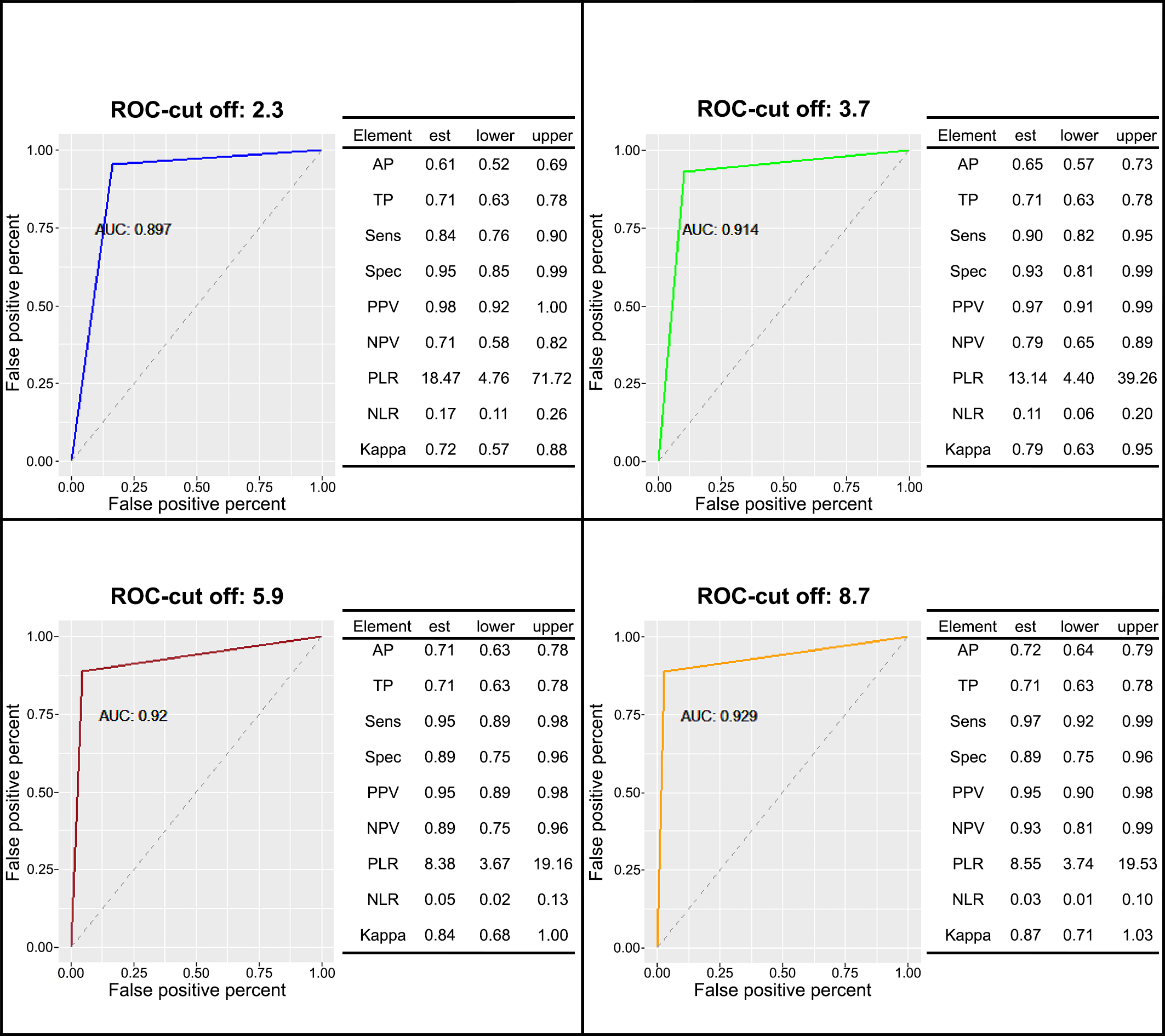

Supplement: Supplementary Figure S1 — Receiver operating characteristic (ROC) curve, area under the curve (AUC) and statistical summary of cutoff point values (2.3, 3.7, 4.8, 5.9 and 8.7). [file Image_1.tif]
